# Secure Collaborative Platform for Health Care Research in an Open Environment: Perspective on Accountability in Access Control

**DOI:** 10.2196/37978

**Published:** 2022-10-14

**Authors:** Giluk Kang, Young-Gab Kim

**Affiliations:** 1 Department of Computer and Information Security, and Convergence Engineering for Intelligent Drone Sejong University Seoul Republic of Korea

**Keywords:** blockchain, attribute-based encryption, eHealth data, security, privacy, cloud computing, research platform for health care, accountability, Internet of Things, interoperability, mobile phone

## Abstract

**Background:**

With the recent use of IT in health care, a variety of eHealth data are increasingly being collected and stored by national health agencies. As these eHealth data can advance the modern health care system and make it smarter, many researchers want to use these data in their studies. However, using eHealth data brings about privacy and security concerns. The analytical environment that supports health care research must also consider many requirements. For these reasons, countries generally provide research platforms for health care, but some data providers (eg, patients) are still concerned about the security and privacy of their eHealth data. Thus, a more secure platform for health care research that guarantees the utility of eHealth data while focusing on its security and privacy is needed.

**Objective:**

This study aims to implement a research platform for health care called the health care big data platform (HBDP), which is more secure than previous health care research platforms. The HBDP uses attribute-based encryption to achieve fine-grained access control and encryption of stored eHealth data in an open environment. Moreover, in the HBDP, platform administrators can perform the appropriate follow-up (eg, block illegal users) and monitoring through a private blockchain. In other words, the HBDP supports accountability in access control.

**Methods:**

We first identified potential security threats in the health care domain. We then defined the security requirements to minimize the identified threats. In particular, the requirements were defined based on the security solutions used in existing health care research platforms. We then proposed the HBDP, which meets defined security requirements (ie, access control, encryption of stored eHealth data, and accountability). Finally, we implemented the HBDP to prove its feasibility.

**Results:**

This study carried out case studies for illegal user detection via the implemented HBDP based on specific scenarios related to the threats. As a result, the platform detected illegal users appropriately via the security agent. Furthermore, in the empirical evaluation of massive data encryption (eg, 100,000 rows with 3 sensitive columns within 46 columns) for column-level encryption, full encryption after column-level encryption, and full decryption including column-level decryption, our approach achieved approximately 3 minutes, 1 minute, and 9 minutes, respectively. In the blockchain, average latencies and throughputs in 1Org with 2Peers reached approximately 18 seconds and 49 transactions per second (TPS) in read mode and approximately 4 seconds and 120 TPS in write mode in 300 TPS.

**Conclusions:**

The HBDP enables fine-grained access control and secure storage of eHealth data via attribute-based encryption cryptography. It also provides nonrepudiation and accountability through the blockchain. Therefore, we consider that our proposal provides a sufficiently secure environment for the use of eHealth data in health care research.

## Introduction

### Background

The development of modern technologies such as the Internet of Things (IoT), cloud computing, big data, and blockchain affects many aspects of human life. Primarily, these technologies have introduced changes in health care. The quality of health care services and operations has also improved because of the digitization of the health care system. Furthermore, with the advancement in sensors, the eHealth data generated by IoT devices for health care are increasingly being collected by health facilities and national health agencies. These eHealth data generally include electronic medical records (EMRs) and personal health records (PHRs), which contain a considerable amount of personal information such as any disease a patient may have and the patient’s medical record number. Thus, some eHealth data subjects have expressed security and privacy concerns related to the use of eHealth data. For this reason, the use of eHealth data is currently governed by many legal regulations, including the Health Insurance Portability and Accountability Act [1], General Data Protection Regulation (GDPR) [2], and California Consumer Privacy Act [3]. However, the security of eHealth data has frequently been breached, and the number of cyberattacks launched to hijack eHealth data intended for health care services is on the rise [4].

**Figure 1:**
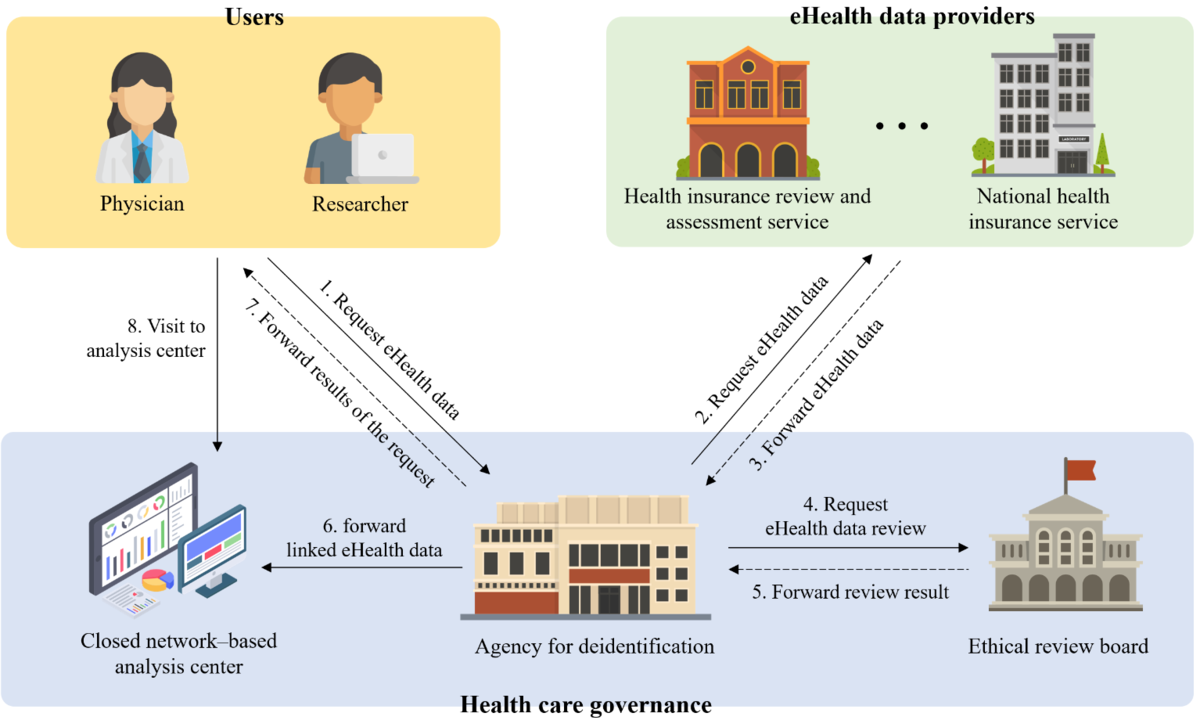
Access procedure for analyzing eHealth data in South Korea.

Nevertheless, using eHealth data for health care research has many advantages (eg, improving treatment and prescriptions for patients, increasing the efficiency of health care systems, and expanding knowledge of diseases), so many researchers hope to use them for their studies [5]. However, the interoperability, utility, and data linkage of eHealth data as well as privacy laws (eg, Health Insurance Portability and Accountability Act, GDPR, and California Consumer Privacy Act) and analytics tools must be considered when a research platform for health care is built. Furthermore, security and privacy measures (eg, anonymization and access control) for an open research environment for eHealth data are needed, and many privacy laws must be complied with. Owing to these complex requirements, most research platforms for health care development are being led by national governments. For example, as depicted in Figure 1, the Ministry of Health and Welfare of South Korea [6] operates a closed network–based analysis center that supports a research environment for analyzing eHealth data. However, researchers must visit the analysis center as they are not able to connect to it remotely or on the web. Not only is this analysis center inconvenient to visit, but it also presents a challenge to efficiently analyzing eHealth data as programming errors can only be corrected via books because of the closed nature of the network. Moreover, the eHealth data requested by the researchers are immediately deleted after use, which reduces the utility of the data.

The National Health Service (NHS) in England also offers eHealth data to researchers and clinicians through a Data Access Request Service (DARS) [7]. The NHS DARS provides various analytical tools such as Databricks, R Studio, and Hue in the data access environment, and it does not require the researcher to visit the research analysis center, unlike the center in South Korea. The NHS DARS also provides many security solutions (eg, 2-factor authentication, data-sharing audits, and anonymization) to ensure the security and privacy of eHealth data. Furthermore, the Swiss Personalized Health Network offers a secure infrastructure for the exchange and use of eHealth data for research [8]. In the Swiss Personalized Health Network, eHealth data can be accessed only from reliable hospitals and universities or the virtual private network, which are environments. Researchers must take the web-based ethics training and are required to complete 2-factor authentication. However, data subjects (ie, patients) are still concerned about unauthorized data reuse and sharing, and they hope to be involved in eHealth data access decisions [9]. In addition, even if eHealth data are deidentified and anonymized, reidentification is still possible via other big data [10,11]. In other words, studies on health care research platforms are needed to provide a more secure analytical environment in light of the apprehension of data subjects regarding the security and privacy of their eHealth data.

Therefore, we propose a secure research platform for health care, referred to as the health care big data platform (HBDP). In this study, we considered only a secure and open research environment, although a research platform for health care has many requirements. The HBDP uses a private blockchain to provide a decentralized persistent log database (DB) in which user activities on the platform are recorded with a time stamp by a smart contract. This helps the platform administrator conduct the appropriate follow-up and monitoring of security threats. Furthermore, the HBDP uses attribute-based encryption (ABE) to ensure the security and privacy of eHealth data and prevent eHealth data leakage by insiders. To the best of our knowledge, this is the first study on a secure research platform that is focused on accountability to secure the use of eHealth data in an open environment based on blockchain and ABE. The main contributions of this study are summarized in Textbox 1.

**Main contributions of this study.**

**Main contributions**

- We propose the health care big data platform (HBDP), which supports the accountability, access control, and encryption of stored eHealth data using attribute-based encryption and a private blockchain in an open environment. In particular, we focused on accountability in access control. We also analyzed previous research platforms for health care from a security perspective.
- For accountability in access control, a smart contract is designed to record in real time the success or failure of user activities (eg, log-in and use of eHealth data) on the HBDP. In particular, the contract enables user monitoring and illegal user detection in the HBDP anytime.
- To prove and demonstrate the feasibility of the HBDP, we implemented a framework for the HBDP using Hyperledger Fabric (The Linux Foundation) [12], OpenABE library (Zeutro) [13], and OpenStack (Open Infrastructure Foundation) [14], and we evaluated its security by using case studies on the detection of illegal users.

### Prior Work

#### Overview

To analyze prior work, we first collected and analyzed well-known health care standards for the interoperability of eHealth data. After analyzing the standards, we searched existing health care studies related to the blockchain using the terms “blockchain” AND “access OR data sharing OR access control” AND “healthcare” for literature review in IEEE Xplore, Wiley Online Library, ScienceDirect, and MDPI. The results identified 501 papers in IEEE Xplore, 943 articles in the Wiley Online Library, 2599 articles in ScienceDirect, and 24,219 articles in MDPI. To select suitable studies, we added some filters (ie, published from 2018 to 2022 and cited by ≥5 journals) based on these results. We also reviewed the abstracts and titles of the papers. On the basis of these works, we finally selected 9 papers (ie, IEEE Xplore: n=4, 44%; Wiley Online Library: n=2, 22%; ScienceDirect: n=2, 22%; and MDPI: n=1, 11%).

Furthermore, we searched health care research platforms from 2015 to 2022 using the terms “healthcare research platform” and “clinical research platform” in Google Scholar. The results showed approximately 849,000 and 1,480,000 papers for each keyword, respectively. To identify suitable studies, we also reviewed the abstracts and titles. In particular, we examined the security solutions in each study and finally selected 6 papers. This section analyzes the identified studies via these processes in detail.

#### Standards for Interoperability of eHealth Data

For a long time, eHealth data have been limited to being shared and accessed between health care providers owing to interoperability issues such as differences in representation (eg, vocabularies and terminologies), equipment, and data formats. These issues currently make it difficult for health care providers to ensure continuity of care for patients or analyze eHealth data in health care. Therefore, many health care organizations are publishing interoperability standards for eHealth data in health care. Clinical Document Architecture (CDA) [15] is an XML-based markup standard for clinical document exchange designed by Health Level 7 (HL7). CDA prescribes the structure and semantics of clinical documents for interaction between health care systems. The central aspect of CDA is easily exchanging clinical documents and making them readable. However, CDA-based documentation has the disadvantage of making it complex and difficult. For this reason, CDA has been extended to Consolidated CDA with improved complexity and interoperability. Fast Healthcare Interoperability Resources (FHIR) [16] is a standard to ensure the interoperability of health care systems or services also developed by HL7. The FHIR improved the limitations of the previously developed HL7 versions 2 and 3 (eg, implementation complexity and structured data model) to make the exchange of medical information easier. Furthermore, it was developed based on the representational state transfer architecture, so it is easy to implement health care services for mobile phones, wearable devices, and tablet devices beyond computers. Thus, the FHIR is currently one of the most popular standards for the interoperability of eHealth data in health care. The Observational Medical Outcomes Partnership (OMOP) Common Data Model (CDM) [17] is an open community standard for the eHealth data model managed by Observational Health Data Sciences and Informatics. The OMOP CDM solves the interoperability issues of eHealth by structuring the data model and the content of observational data. The OMOP CDM structures eHealth data to provide a common data model and converts them into a common representation through the OMOP to provide the common physical and logical interoperability model. When a health care DB is designed via the OMOP CDM, it can use standardized analysis tools and help analyze eHealth data systematically. It also increases the efficiency of joint research. Digital Imaging and Communications in Medicine (DICOM) [18] is a data format standard for the interoperability of medical imaging such as magnetic resonance imaging, computed tomography, and x-rays. DICOM has defined the format of medical imaging so that medical images captured by various imaging devices can be transmitted and exchanged. DICOM is generally stored, processed, and transmitted via the picture archiving and communication system and is the best known today in health care. Cross-Enterprise Document Sharing (XDS) [19] is an integrated profile for eHealth data developed by Integrating the Healthcare Enterprise in 2004. In particular, XDS can share various standard-based clinical documents such as the HL7 CDA, general strings, and binary data. In other words, XDS represents a comprehensive and universal technology. In addition to the aforementioned standards, various standards are being established for the interoperability of eHealth data by many health care organizations. We consider that these standards do not provide perfect interoperability of eHealth data but can still be addressed in the near future. Thus, the interoperability of eHealth data is the main requirement in the research platform for health care, but it is not the main focus of this study.

#### Secure eHealth Data Sharing via Blockchain

The blockchain has many advantages (eg, data integrity, decentralization, and programmable smart contract), so many research areas have been trying to use it. In particular, the blockchain has been widely used to address the integrity, scalability, and sharing of eHealth data. However, in addition, eHealth data require security mechanisms such as access control, cryptography, and authentication owing to privacy and security issues. For this reason, many studies generally use these security mechanisms with the blockchain. Table 1 shows the strengths and weaknesses of these studies and the HBDP. Yang et al [20] proposed an architecture that can use blockchain in the existing health care system. The architecture has recorded all accesses, such as select, insert, and delete, using two smart contracts (ie, summary contract and record relationship contract) to ensure the integrity of data records. The architecture also performs access control via an access control list. Madine et al [21] proposed a blockchain-based, patient-centric PHR management system. The system uses trusted oracles that perform proxy re-encryption to share the PHRs securely. Furthermore, the system uses a reputation system to track an oracle’s behavior and give a rating score to identify the misbehaving oracles. Thus, the system lets them fetch, store securely, and share medical data. Zhang et al [22] presented the architecture for sharing clinical data based on blockchain. The architecture used the FHIR standard and blockchain to solve clinical data interoperability and is called FHIRChain. The FHIRChain helps enable collaborative clinical decision-making among physicians. It also allows for the sharing of clinical data in a trustless and decentralized environment and for auditing through the smart contract. Shahnaz et al [23] designed the role-based access control (RBAC) framework for EMRs using smart contracts. They focused on solving the scalability problem of blockchain via the off-chain scaling mechanism.

Tanwar et al [24] proposed a permission-based system architecture that could share eHealth data using blockchain. In this architecture, patients can join the blockchain network through the client application and update their eHealth data on the blockchain network via chain code. They can also grant or revoke permission to clinicians and researchers for their eHealth data. In conclusion, the architecture achieves patient-centric eHealth data sharing. Figueroa et al [25] used attribute-based access control for the security of a radio frequency identification system for health care. They focused on solving system problems such as scalability, synchronization, and single point of failure using blockchain. Ultimately, the system offers access control to use the medical assets from a suitable location. Daraghmi et al [26] designed a blockchain-based EMR management system called MedChain. They improved the block time and system performance using proof of authority. They also used time-based smart contracts for the privacy and monitoring of EMRs. In brief, they provided a secure environment, data integrity, auditability, and accessibility using authentication techniques, hash function, and proxy re-encryption. Kaur et al [27] proposed blockchain-based storage for securely sharing and querying eHealth data. The storage uses CouchDB considering the unstructured eHealth data. It also stores EMRs in the off-chain and hash of EMRs on the blockchain to ensure the integrity of EMRs and improve the efficiency of storage. Guo et al [28] proposed the multi-authority ABE scheme for cloud-based telemedicine systems. In particular, the scheme protects the integrity of eHealth data (eg, diagnostic opinions) using the blockchain. Furthermore, the scheme updates and revokes the access policy easily.

Most studies [20-28] only focused on blockchain for secure sharing and ensuring the integrity of eHealth data among hospitals. They generally mentioned traceability and accountability via the blockchain, but they did not represent methods for monitoring and accountability. However, these methods should be presented to ensure a secure environment. In particular, accountability is essential in a health care research platform in open environments. For these reasons, unlike other studies, the HBDP focused on the description of the detection method based on the blockchain to ensure accountability. In addition, in the HBDP, even if eHealth data are exported, the data are not ensured usability as they can only be decrypted and used in the HBDP. As mentioned previously, this study is the first to focus on accountability to use eHealth data in an open environment securely.

**Table 1:** Strengths and weaknesses of blockchain-based studies and the health care big data platform (HBDP).

Studies	System name	Security solutions	Strength	Weakness
Yang et al [20]	—^a^	Encryption Access control	Interoperability among existing health care systems	The proposed architecture only focused on reading health records and did not discuss sharing of health records.
Madine et al [21]	—	Encryption Blockchain	A patient-centric PHR^b^ management system is proposed.	Not useful for an emergency where the patient is not able to delegate permission
Zhang et al [22]	FHIRChain	Audit Access control	No SPoF^c^ problem and fine-grained access control	The architecture only presented the possibility of health data tracking.
Shahnaz et al [23]	—	Access control	The proposed architecture solves the scalability problem of blockchain via off-chain scaling.	The proposed architecture requires transaction costs and fees for access control.
Tanwar et al [24]	—	Access control	A patient-centric eHealth data sharing is achieved.	Lack of flexible and fine-grained access control
Figueroa et al [25]	—	Access control	No SPoF problem and fine-grained access control	The architecture requires transaction costs and fees for access control.
Daraghmi et al [26]	MedChain	Encryption Authentication	Efficient consensus mechanism and ensuring privacy via time-based smart contracts	No detailed description of the implementation of the proposed system using the PoA^d^
Kaur et al [27]	—	Authorization	Sharing of unstructured eHealth data and off-chain storage	Not useful for an emergency where the patient is not able to delegate permission
Guo et al [28]	—	Encryption Access control	The ABE^e^ scheme is proposed as suitable for the distributed telemedicine system.	The specific method is not presented to ensure the traceability of the schema.
Ours	HBDP	Encryption Audit Access control	The detailed methods for accountability in access control are proposed.	The platform focuses only on 3 SRs^f^.

^a^Not presented.

^b^PHR: personal health record.

^c^SPoF: single point of failure.

^d^PoA: proof of authority.

^e^ABE: attribute-based encryption.

^f^SR: security requirement.

#### Health Care Research Platforms

The use of eHealth data in health care research can fundamentally improve health care owing to the rapid development of big data analytical technologies. For this reason, several studies have proposed health care research platforms that can be used for research using eHealth data. In this section, we review the literature with a focus on the security perspective of these research platforms. Ozaydin et al [29] proposed the design of a data warehouse, which is a Healthcare Research and Analytics Data Infrastructure Solution (HRADIS). The HRADIS focuses on infrastructure for integrating disparate eHealth data to improve the efficiency of health care. The HRADIS includes an account management framework for RBAC for some eHealth data. Lunn et al [30] proposed a cloud-based digital health research platform for a national longitudinal cohort study. The platform collects and manages eHealth data of sexual and gender minority adults. In this platform, all microservices are within the subnet using virtual private cloud, and eHealth data at rest are stored in the MySQL DB securely after encryption. Furthermore, the platform uses open authorization for programming interfaces, SMS text message–based 2-factor authentication, and logging services to identify malicious users and ensure the security of eHealth data. Ashfaq et al [31] described the regional health care information platform in Halland, Sweden. The platform basically operates within Swedish regulations and the GDPR regarding patient data. On the platform, eHealth data can only be accessed through internal clients secured in the regional IT firewalls. The client can only use related researchers in the approved health care project via the ethical review board in Sweden. In particular, the platform provides anonymized eHealth data to ensure privacy. Conde et al [32] presented an open source–based research platform to support clinical and translational studies, ITCBio. The ITCBio platform supports role and access management tools to promote research collaboration and ensure security. It also provides dynamic consent, which enables ongoing and flexible communication between patients and researchers. De Moor et al [33] described a scalable and adaptable platform for the interoperability of eHealth data systems and clinical research systems. They also presented the security architecture based on many security-related standards in detail. In particular, this architecture supports various security solutions such as identity management and credential delegation. Jones et al [34] proposed the Secure Anonymised Information Linkage databank, which is ensured physical, technical, and procedural control. The Secure Anonymised Information Linkage databank provides encrypted communication and prevents eHealth data from being transferred outside the user’s devices. It also performs user authentication via user credentials and 2-factor authentication tokens.

Several studies [29-34] have proposed health care research platforms for using eHealth data. However, most studies have focused on an efficient research environment. Some studies also did not describe security solutions in detail despite the security and privacy of eHealth data being major considerations in health care research platforms. Moreover, as mentioned previously in the Background section, eHealth data subjects are still concerned about the security and privacy of eHealth data. Thus, a study is necessary for a more secure platform for health care research that guarantees the usability of eHealth data while focusing on its security and privacy. The next section proposes a secure and expandable collaborative research platform for health care called the HBDP.

## Methods

### Overview

This study designed a secure and open environment for health care research. To accomplish this, we first identify potential security threats on a health care research platform. Second, we propose security requirements (SRs) for a secure health care research platform based on these threats. Finally, we present a secure collaborative research platform for health care called the HBDP that can provide a secure analysis environment while meeting these requirements.

### Security Threats and Requirements on a Health Care Research Platform

#### Overview

A health care research platform should properly understand and mitigate security threats to provide a secure analytical environment. This subsection first identifies potential security threats of health care research platforms. We then define the SRs for mitigating these threats.

#### Security Threats

Various security threats, such as the abuse and illegal export of eHealth data, can arise on a health care research platform. However, we identified well-known security threats in the health care domain as threats to the health care research platform. In other words, many threats can occur on the platform, but we explicitly focused on threats that can occur frequently. A detailed description of the leading security threats is outlined in Textbox 2.

**Leading security threats on a health care research platform.**

**Leading security threats**

- Unauthenticated users: on a health care research platform, unauthenticated users attempt an attack to obtain the authenticated user’s credentials [35-37]. In addition, attackers can invalidate the authentication factor to access eHealth data [38]. Hence, a health care research platform must ensure, through user authentication, that only authenticated users have access.
- Unauthorized users: a health care research platform must ensure that only approved eHealth data are available to authorized users through appropriate authorization mechanisms [38,39]. Moreover, the abuse and illegal sharing of eHealth data can occur on a health care research platform even by authorized users. Therefore, a health care research platform also requires a security solution that audits for these activities.
- Leaks of eHealth data by insiders: the greatest security threat for a health care research platform is a breach of eHealth data by insiders [35-37,40]. A prime example of an insider is the eHealth data administrator of the health care research platform. The administrator can easily leak eHealth data as they have general authorization over them. Furthermore, insiders are difficult to detect as they are defined as suitable users within the health care research platform. For these reasons, even if eHealth data on a platform are illegally leaked, the utility of leaked data must not be ensured.

#### SRs for Mitigating These Threats

A collaborative health care research platform in an open environment should satisfy the diverse SRs that mitigate many types of security threats. However, in this study, we only focused on 3 SRs, which are highly related to accountability in access control for a secure health care research platform based on the aforementioned identified threats. The detailed descriptions of the SRs are outlined in Textbox 3.

**Security requirements (SRs) for mitigating security threats.**

**SRs for threat mitigation**

- SR 1 (access control): access control is a framework that includes authentication and authorization, which is the primary SR and the most important consideration for a health care research platform. It must be performed on this platform so that only authenticated and authorized users can use eHealth data via appropriate devices. For this reason, many existing health care research platforms provide authentication or authorization using various methods [29-34].
- SR 2 (encryption of stored eHealth data): on a health care research platform, the encryption of stored eHealth data ensures the security and privacy of eHealth data when the data are not being used [30,34]. In addition, even if eHealth data are leaked, the data should not be useful. Hence, the encryption of stored eHealth data is one of the most important SRs.
- SR 3 (accountability): when the authenticated and authorized user exports or uses eHealth data via the research platform for health care, the platform administrator or eHealth data provider needs to be able to track and search all the user’s activities on the platform at any time. In addition, the platform administrator must identify illegal users and conduct the appropriate follow-up or monitoring in the event of security issues. For these reasons, some health care research platforms provide logging systems or services [30,34].
- Other SRs: the collaborative research platform in an open environment should satisfy various other SRs. For example, anonymization and deidentification are needed for the privacy of eHealth data as the data are sensitive and private [31,32,34]. Secure communication is also necessary to prevent sniffing and tampering with eHealth data and network packets [30,31,34]. In addition, more SRs for the integrity and availability of eHealth data are required [41]. However, as mentioned previously, we focused on the three SRs (ie, access control, encryption of stored eHealth data, and accountability) to support accountability in access control.

### Proposed HBDP

#### Overview

The HBDP uses ABE for the privacy and access control of eHealth data. In particular, the privacy of eHealth data is ensured through column-level encryption even if insiders leak the data. The platform also uses a smart contract to record user activities (eg, log-in and decryption) in the blockchain. Thus, the blockchain allows platform administrators to identify illegal users and conduct appropriate follow-up and monitoring. In other words, the blockchain operates as a distributed logging system in real time and ensures the integrity and nonrepudiation of recorded user activities. In this section, to present the HBDP, we first explain the assumptions and main components in a framework. We then describe the phases of the HBDP in detail.

#### Assumptions

To describe a framework and scenarios of HBDP, we first define some assumptions. In particular, we present assumptions about other SRs (eg, secure communication, deidentification, integrity, and availability) that the HBDP does not cover. The detailed assumptions are outlined in Textbox 4.

**Assumptions about other security requirements.**

**Assumptions**

- As eHealth data contain a considerable amount of personally identifiable information, they are generally provided to users after deidentification and anonymization on the platform. Data linkage is also performed to increase the usability of eHealth data before they are provided to users. However, this study did not cover deidentification, anonymization, and data linkage. Thus, all the eHealth data on the platform are assumed to be deidentified and linked via trusted third-party organizations. In addition, eHealth data are assumed to be provided by institutions registered on the platform.
- This study did not cover secure communication between the health care big data platform (HBDP) and the users. Therefore, we assume that the HBDP is securely communicating with its users by using transport layer security protocol–based communication, which is used for secure communication on the internet and across networks. This assumption also holds for communication on the blockchain network.
- Users are assumed to be researchers or physicians with a specific institution that is registered on the HBDP. Thus, they do not need to prove that they are researchers or physicians affiliated with the institution when they register on the platform, but authentication and authorization for access to eHealth data for users are performed on the platform. Furthermore, we also assume that the HBDP provides a variety of analytical tools and methods for researchers to efficiently analyze eHealth data and that the user analysis process is recorded on the distributed ledger, including the analytical tools used.

#### Main Components

**Figure 2:**
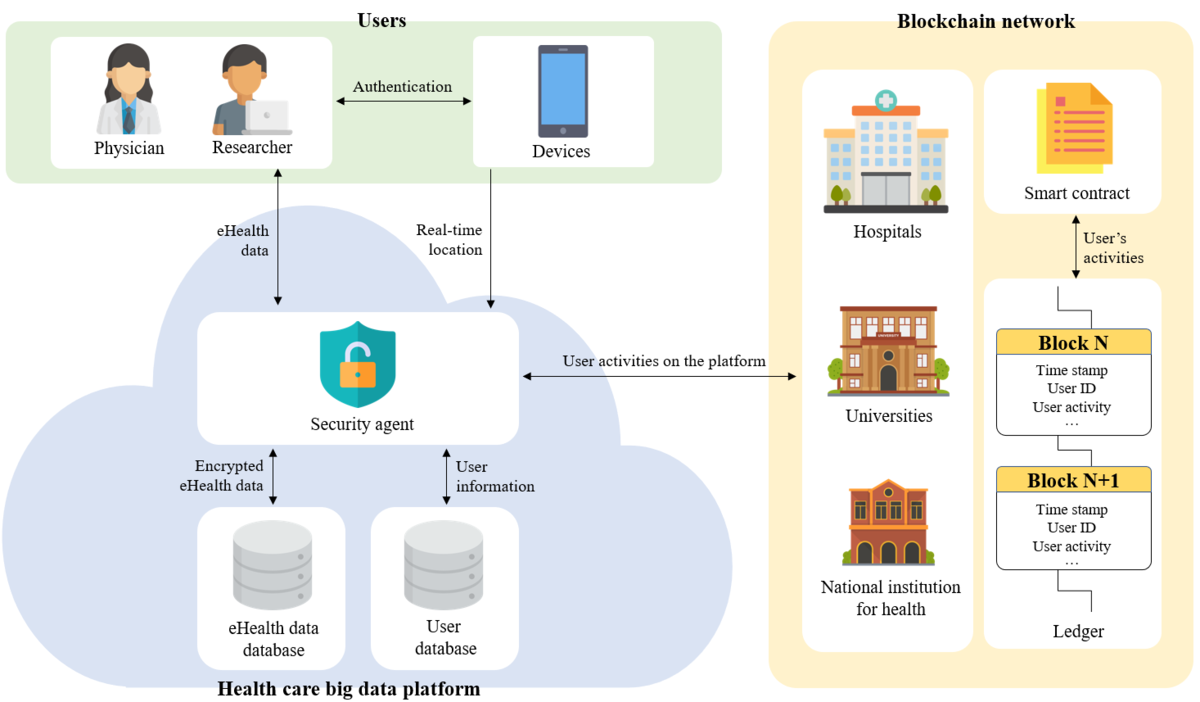
Overview of our proposed framework for the health care big data platform.

We present a secure collaborative platform for health care research that ensures the privacy and security of eHealth data, called the HBDP. Figure 2 shows a brief overview of our proposed framework for the HBDP. Our proposed framework has 3 main components: users, the HBDP, and the blockchain network. A detailed description of the main components is outlined in Textbox 5.

**Main components of our proposed framework.**

**Framework components**

- Users: physicians and researchers who analyze and use eHealth data to treat patients or use them for health care research are representative of this group. They should be required to have a device such as a smartphone or a fingerprint scanner with a GPS for authentication and access control on the platform.
- Health care big data platform (HBDP): the HBDP keeps eHealth data secure and provides an environment where users can use and analyze the data. The platform consists of a security agent and databases (DBs) in a cloud computing environment. DBs are configured as eHealth DBs and user DBs. The eHealth data DB stores eHealth data. The user DB stores user information such as the user ID, hashed password, and user attributes (eg, user department and position). The security agent is a key component of the platform. It performs encryption and decryption of eHealth data using attribute-based encryption. It also requests, as a blockchain client, the blockchain network to record or obtain user activities.
- Blockchain network: the blockchain network consists of a single smart contract, a distributed ledger, and peers. The transactions recorded on the blockchain network are immutable unless the ledgers of all peers are modified. For this reason, the blockchain can be used as a distributed logging system that provides strong accountability, so we use the blockchain network for the tracking of user activities on the HBDP. More specifically, the blockchain communicates with the security agents on the HBDP and helps ensure the accountability and nonrepudiation of the platform. Peers are health facilities and research institutes registered on the platform. They can be endorsing peers or committing peers depending on their system performance. The smart contracts record user activities with time stamps on the distributed ledger, which helps the distributed ledger in the blockchain act as logs for the HBDP.

#### Phases of the HBDP

##### Overview

To support secure analytical environments, the HBDP has 4 phases (ie, user registration, storage, download, and use). Each phase is configured to satisfy our defined SRs (ie, the user registration and download phases meet the access control requirement, the storage phase meets the encryption of stored eHealth data requirement, and the use phase achieves accountability). A detailed description of each phase is provided in the following sections.

##### User Registration Phase

**Figure 3:**
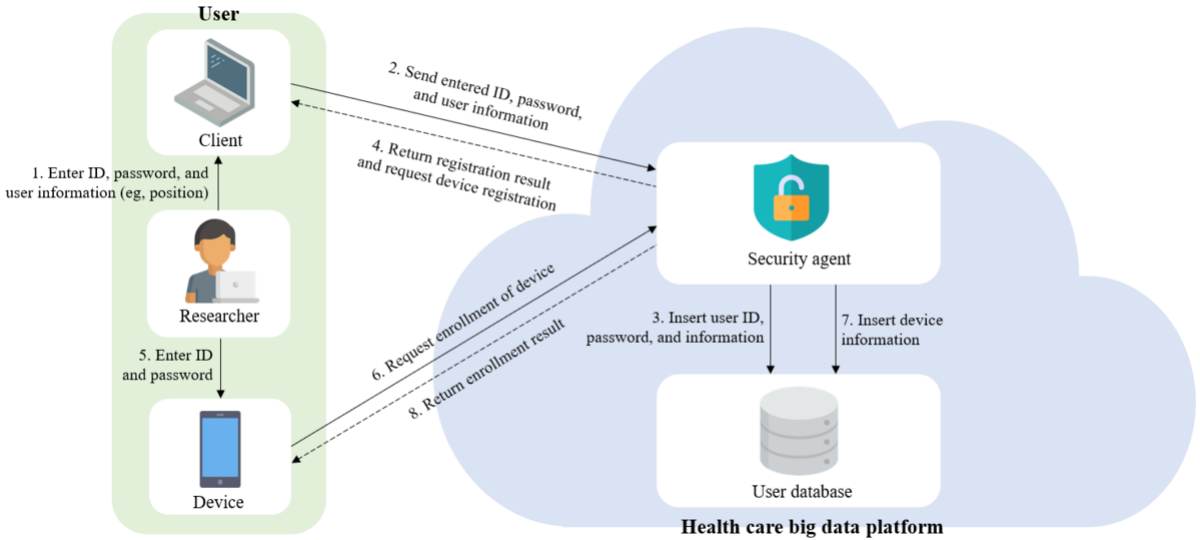
User registration phase.

The user registration phase is the first operation for authentication in “access control,” which is one of the SRs of a health care research platform. This phase is stored with the user ID and attributes in the user DB on the HBDP. Figure 3 shows a sequence of the user registration phase; the details are described in this section. The user accesses the HBDP and enters the user ID, password, and attributes (eg, the user’s department and position). At this time, we assume that this user is authorized by an institution participating in the HBDP. The security agent on this platform then inserts the entered user information into the user DB. The security agent requests device enrollment from the user with the registration result. The user accesses the platform using their device and enters the registered ID, password, and device identifier. The user device then requests the security agent to enroll it along with the entered ID and password. The security agent performs password-based authentication using the received ID and password. If this authentication is successful, the device ID value is inserted into the user DB, and the security agent relays the result of the device enrollment to the device. After that, the user can access the HBDP at any time.

##### Storage Phase

**Figure 4:**
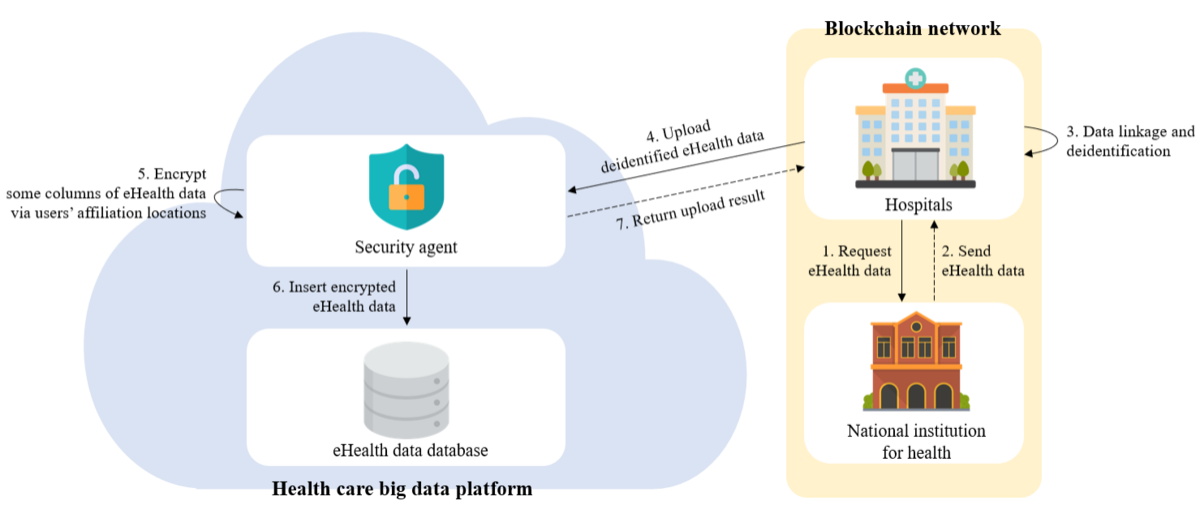
Storage phase.

Attempts to leak eHealth data stored by the HBDP may frequently occur. Even if eHealth data are inevitably leaked in these attempts, the nonusability of the data should be ensured. For this reason, in the storage phase, as shown in Figure 4, the security agent encrypts eHealth data using the locations of institutions registered on the platform before storing the eHealth data on the platform.

In particular, some columns are sensitive columns that provide usability for researchers during the analysis of eHealth data or can be combined with other big data to identify individuals. The storage phase is the operation that ensures the “encryption of stored eHealth data,” which is one of the SRs for health care research platforms. This phase ensures that, even if eHealth data are illegally shared or leaked by the administrator or a malicious attacker on the platform, their usability is not ensured because of column-level encryption. Moreover, column-level encryption allows users to use decrypted eHealth data only at their institutions as the decryption of eHealth data fails if the user’s real-time location does not match the user’s institution.

##### Download Phase

**Figure 5:**
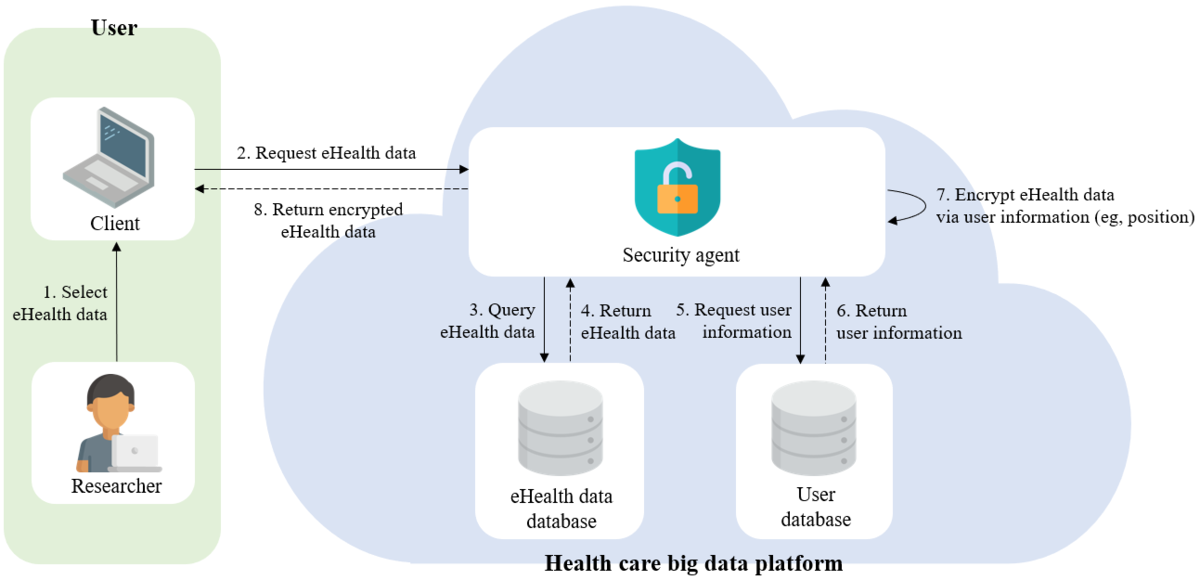
Download phase.

The download phase is the prerequisite for the authorization process in the “access control” for the SRs of the health care research platform. This phase encrypts all contents of column-level–encrypted eHealth data via user attributes (eg, user position and department) to enable the user to download eHealth data. Therefore, the data provided during the download phase are encrypted, so they are impossible to analyze even if a third party obtains them. The download phase is not an essential phase but can increase the efficiency when collaborative research is conducted. For example, if all collaborative researchers are authorized on the platform, the researcher sends eHealth data after the first analysis to another researcher for collaboration. Another researcher can then proceed with further work based on the analyzed eHealth data on the platform. Figure 5 shows the download phase on the platform. A detailed description of the download phase is provided in the following paragraph.

The user first logs into the HBDP and selects the eHealth data on the download page. The security agent on the platform then sends a query for the eHealth data requested by the user to the eHealth data DB. The eHealth data DB provides column-level–encrypted eHealth data to the security agent. After that, the security agent requests user information, such as the user’s attributes and ID, from the user DB. The user DB provides the requested user information to the security agent. The security agent encrypts the column-level–encrypted eHealth data one more time using the provided user information and offers the full encrypted eHealth data. The eHealth data provided during the download phase are encrypted, so they are impossible to analyze even if a third party obtains them.

##### Use Phase

**Figure 6:**
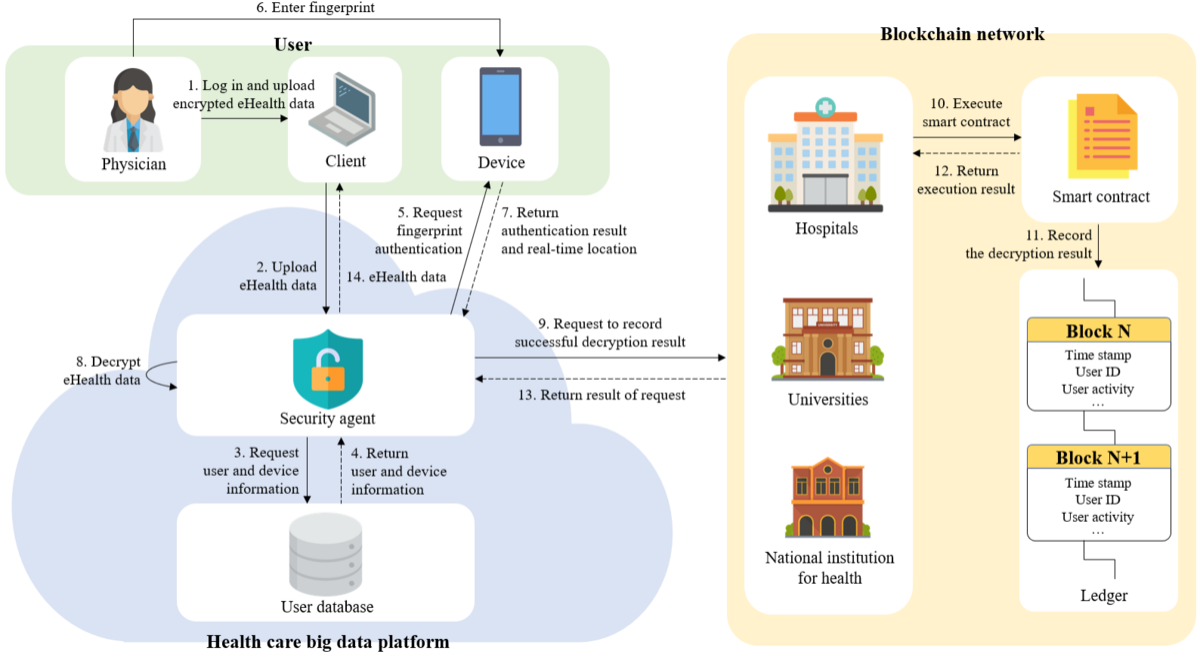
Use phase.

The use phase performs “access control,” which is one of the SRs for the health care research platform. This phase also ensures “accountability” as the use activity of the user on the platform is recorded with a time stamp in the distributed ledger. Figure 6 shows the sequence of the use phase; the details are described in the following paragraphs.

The user logs into the HBDP (ie, password-based authentication) and uploads encrypted eHealth data. The security agent on the platform then requests user and device information from the user DB for decryption and fingerprint authentication. The user DB provides the security agent with the user attributes and user device ID. The security agent uses the device ID value to request fingerprint authentication and real-time location from the user device. The user enters a fingerprint via the enrolled device on the platform for second user authentication. If the user succeeds in fingerprint authentication, the device sends a real-time GPS location to the security agent. The security agent decrypts the eHealth data uploaded by the user using the location and user attributes. In particular, at this time, full decryption is performed on encrypted rows, and column-level decryption is then performed on column-level–encrypted columns (ie, sensitive columns). After the decryption of the eHealth data, the security agent requests the blockchain peer to record the decryption result with a time stamp in the blockchain. Blockchain peers execute a smart contract to record the decryption result on the distributed ledger, and the smart contract inserts the decryption result on the distributed ledger. When the decryption result is recorded in the distributed ledger, the smart contract returns the execution result to the blockchain peers. The blockchain peers send the received execution result to the security agent. Finally, the security agent provides the decrypted eHealth data to the user, and the user is able to use the data only on the platform. In conclusion, for the user to use eHealth data on the HBDP, the data must be decrypted on the platform.

**Figure 7:**
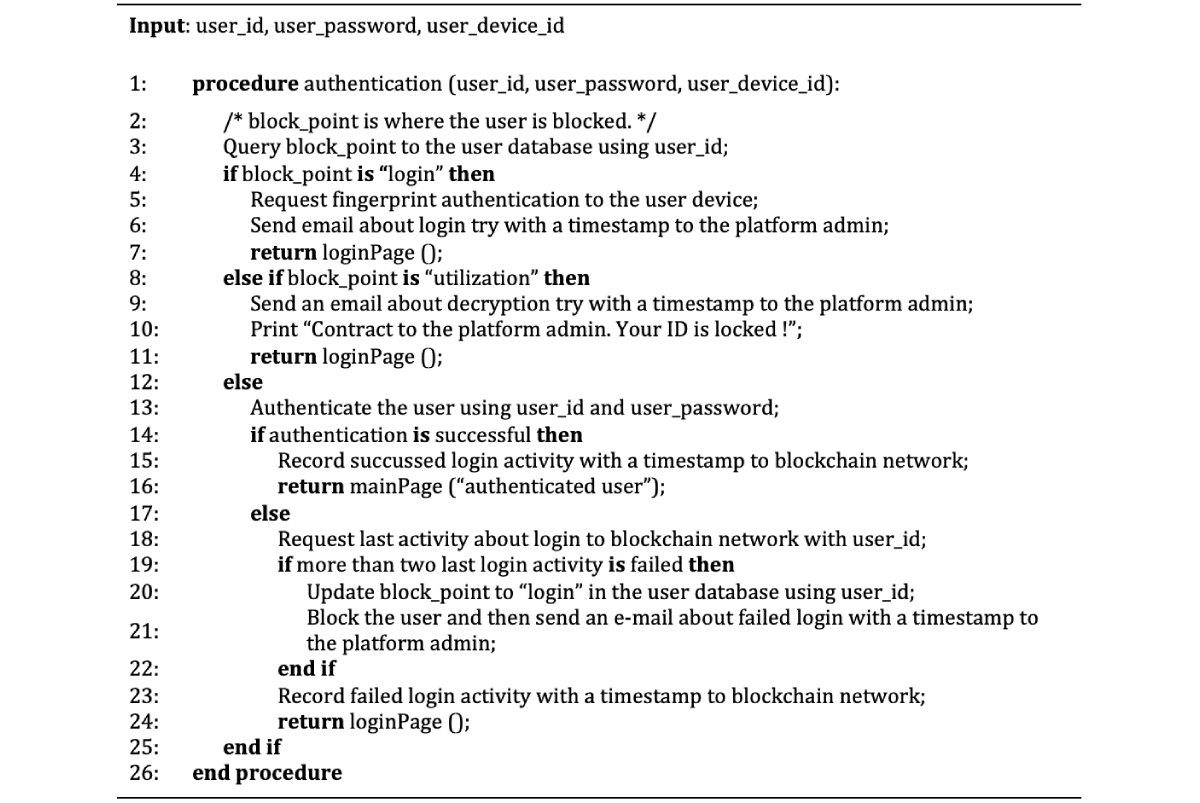
Algorithm 1: pseudocode of authentication for the security agent.

Algorithms 1 and 2 are a pseudocode of the security agent. Algorithms are composed of password-based authentication and decryption of the eHealth data along with the detection of illegal users. In particular, algorithm 1 (Figure 7) first checks whether the user’s ID is locked; if the user’s ID is not locked, it authenticates the user’s credentials. The security agent then requests to record the result of the user log-in activity in the blockchain.

**Figure 8:**
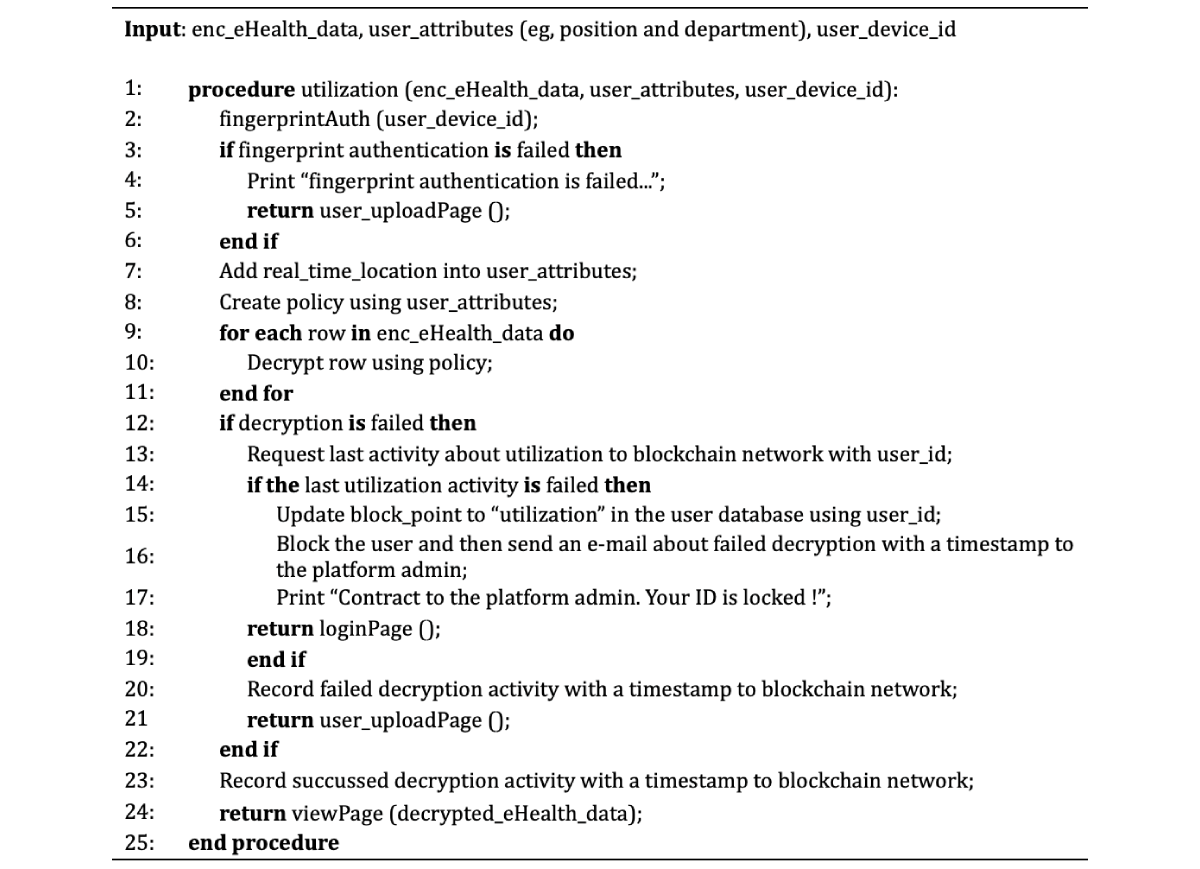
Algorithm 2: pseudocode of use for the security agent.

By contrast, algorithm 2 (Figure 8) first performs fingerprint-based authentication for the user’s real-time location. After that, the security agent decrypts the data using the created policy via user information, including the location. Finally, the security agent requests to record the result of the use activity of the user in the blockchain. In particular, before recording the result from the procedure, the security agent checks the distributed ledger for the most recent consecutive failed activities in the previous records.

#### Implementation

To demonstrate and prove the feasibility of the HBDP, we implemented the main components of a framework based on the software development life cycle. The software development life cycle is generally configured as requirement analysis, design, implementation, testing, and evolution steps. Following these steps, we first identified SRs (see the SRs in the Security Threats and Requirements on a Health Care Research Platform section). Second, the components were designed based on 3 identified SRs (ie, access control, encryption of stored eHealth data, and accountability). Third, we implemented these components. Textbox 6 shows the specifications for the configuration and implementation environment in detail. We configured the blockchain network for detecting illegal users and a cloud environment to create a scalable, collaborative, and secure environment in the HBDP. In particular, we built the cloud environment using OpenStack (Open Infrastructure Foundation), an open-source cloud operating system, and then developed a web server using the Python-based Flask framework (Python Software Foundation) [42]. We also developed an Android app for user authentication, the security agent for detecting illegal users and monitoring, and a chain code to record and manage user activities. Fourth, the components are tested using a security analysis of the HBDP via case studies in the Results section. Finally, the components are analyzed in the Discussion section to evaluate them.

**Figure 9:**
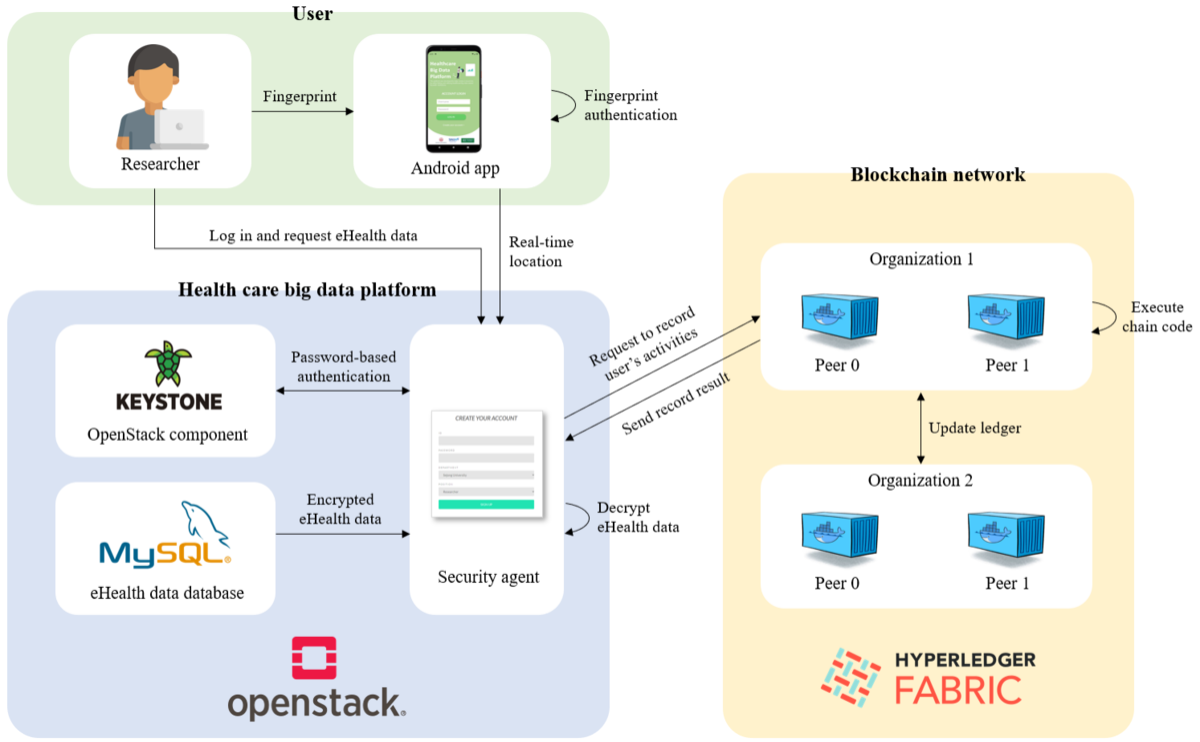
Overview of our implementation.

Figure 9 shows an overview of our implementation and the interactions between the main components. As mentioned previously, our implementation is a proof of concept for demonstrating the features realized by the HBDP.

On a research platform for health care, the cloud environment not only provides various big data analytical tools in the form of software as a service but also provides an environment where researchers can collaborate. The cloud environment also provides scalability and an open environment.

**Figure 10:**
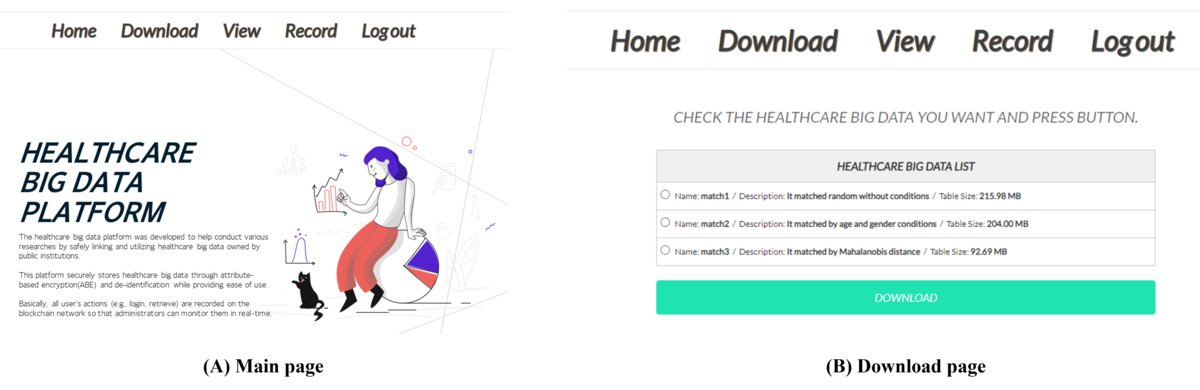
Implemented health care big data platform (HBDP).

Figure 10 shows some pages from the HBDP. Figure 10A is a page that is shown when a user successfully logs in by entering a registered user ID and password. Users who successfully log in can access this platform at any time and download eHealth data that can be used on the page shown in Figure 10B.

**Specifications of our implementation environment.**

**Environment specifications**

- Processor: Intel Xeon processor E5620 2.40 GHz
- Memory: 32 GB
- Operating system: Ubuntu Linux 18.04.5 LTS
- Smartphone: Galaxy S21 (SM-G991N)
- Languages: Go language, Java, Python, and C++
- Docker engine: version 20.10.7
- OpenStack: version 5.2.0 (Stein)
- MySQL: version 5.7.36
- Android: version 11
- Hyperledger Fabric: version 1.4

**Figure 11:**
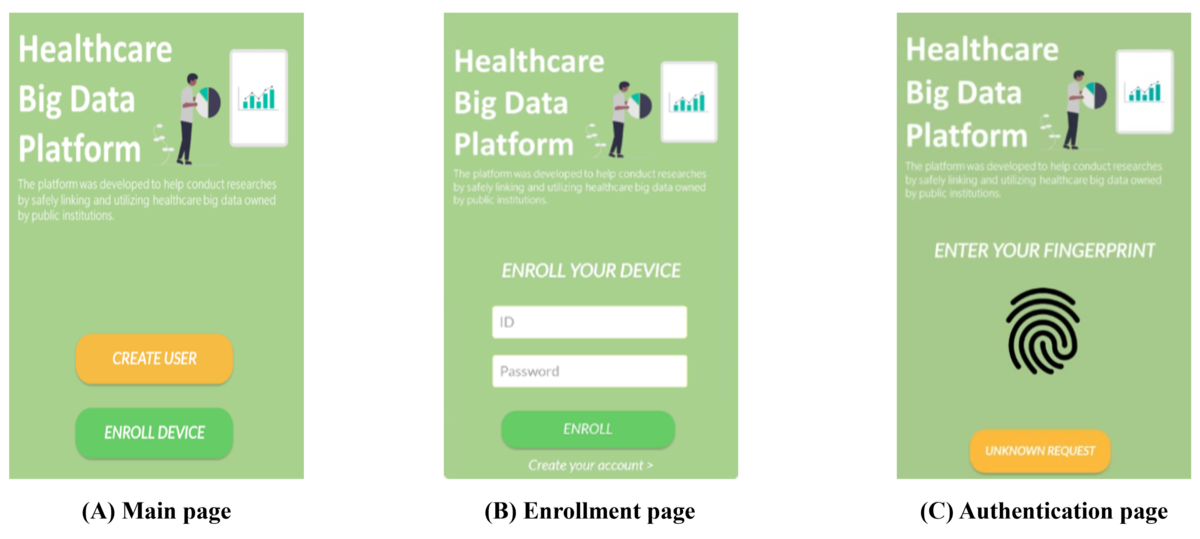
Android app.

We also developed an Android app to use fingerprint authentication and real-time location information. In particular, we used Firebase (Firebase Inc) [43] for messaging the Android app. Figure 11 shows some pages of the implemented app. In Figure 11A, the app performs user registration and device enrollment on the platform. In addition, Figure 11B shows device enrollment for fingerprint authentication and real-time location information. Finally, Figure 11C shows a fingerprint authentication request when the user wants to use encrypted eHealth data.

**Figure 12:**
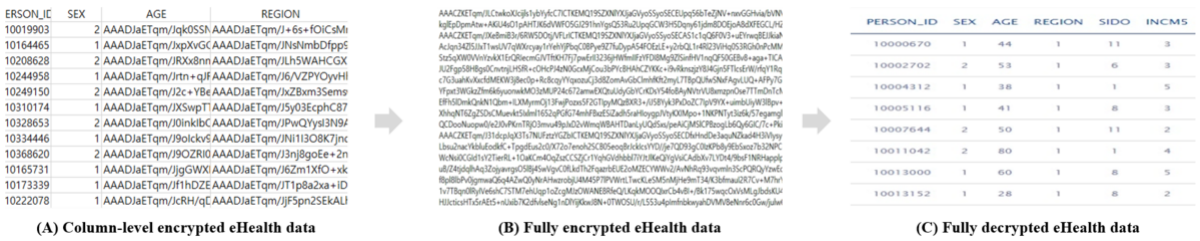
Encryption and decryption of eHealth data in the health care big data platform.

Furthermore, we used the OpenABE library (Zeutro) to perform the encryption and decryption of eHealth data. The eHealth data were anonymous and deidentified open data from the Korea Disease Control and Prevention Agency. The data were from the National Health and Nutrition Examination Survey in South Korea, and they included ID, gender, age, region, and income. In particular, we defined the sensitive columns in our implementation as age and region. Figure 12 shows the eHealth data at each phase on the platform. Figure 12A shows the eHealth data with column-level encryption when they are stored on the platform by the security agent. In addition, when the user downloads eHealth data, they are offered after full encryption of the column-level–encrypted eHealth data using the user attributes stored on the platform, as shown in Figure 12B. Finally, Figure 12C shows the decrypted eHealth data, which can be used by authorized users on the platform.

**Figure 13:**
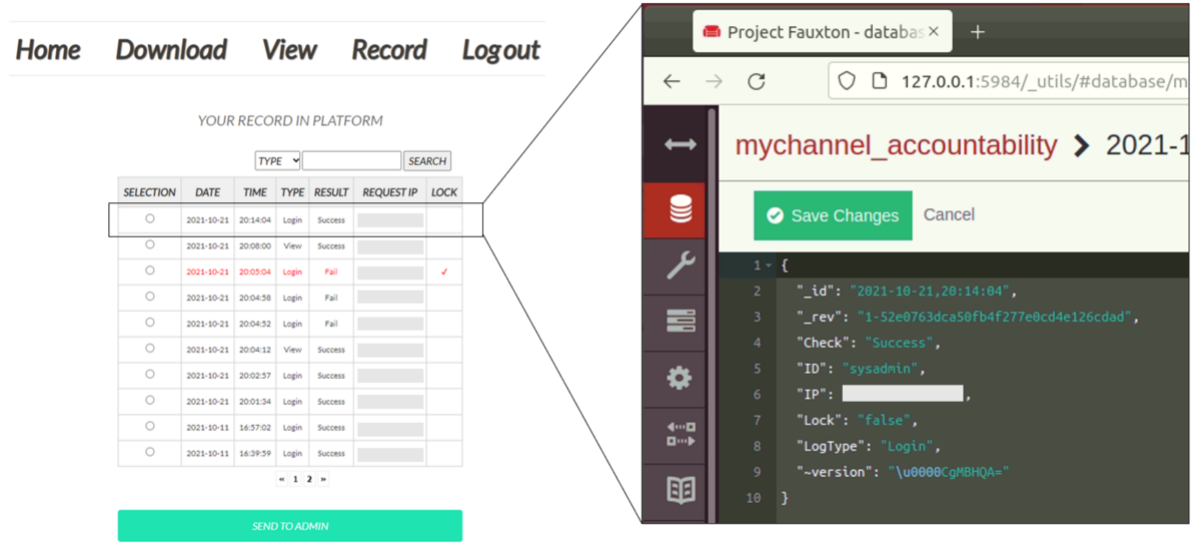
Web page for detection of illegal users.

Generally, blocks in the blockchain are identified by hashes, and the blocks are connected because they have a hash of the previous block. In other words, alteration is impossible unless the blocks of all participants are modified. Therefore, on the platform, the private blockchain is used as a decentralized persistent logging system. We built the blockchain network using Hyperledger Fabric (The Linux Foundation) and designed a smart contract for accountability. Figure 13 shows a web page for detecting illegal users (ie, unauthenticated and unauthorized users) using the distributed ledger. This page helps platform administrators search for specific user activities as well as identify and respond to the actors when security threats arise. In short, the distributed ledger in our implementation provides accountability and nonrepudiation to the HBDP.

## Results

### Overview

To show the proof of concept, the previous section implemented the HBDP. This section describes case studies of illegal user detection for security analysis via the implemented HBDP. This section also presents the results of several conducted experiments, which show the efficiency of the private blockchain and ABE cryptography.

### Case Studies of Detection of Illegal Users

#### Overview

In addition to the aforementioned security threats, many security threats (eg, the misuse and abuse of eHealth data) can arise on a health care research platform in an open environment. Thus, a secure health care research platform must be able to detect and trace the threats. This subsection describes how to detect two representative security threats—unauthenticated and unauthorized users—through the distributed ledger on the HBDP in an open environment. Moreover, we present the possibility of detecting other security threats through monitoring and access control processes.

#### Detection of an Unauthenticated User

**Figure 14:**
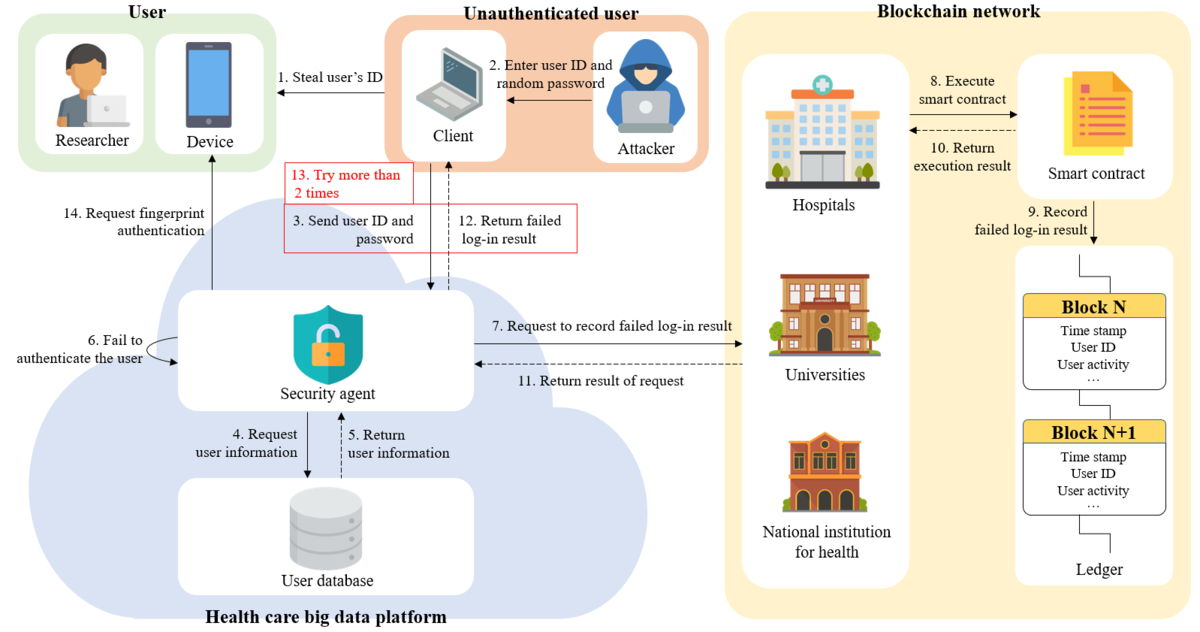
Detection of unauthenticated user.

One of the most common attacks attempted by unauthenticated users is the brute-force attack. Therefore, in this scenario, we assume that an unauthenticated user continuously tries to log into (ie, launches a brute-force attack on) the HBDP by stealing the user ID. Figure 14 shows the sequences for detecting an unauthenticated user on the platform. A detailed explanation of this case study is provided in the following paragraphs.

The attacker first steals the user ID that is used on the HBDP and then connects to the platform and inputs the stolen user ID and a random password. The security agent that receives the user ID and password retrieves the user information from the user DB and performs password-based user authentication. After that, the security agent requests the blockchain peer to record the log-in result in the distributed ledger. The blockchain peer records the log-in result through the execution of a smart contract and sends the recorded result to the security agent. The security agent receives the recorded result and then informs the attacker of the log-in failure. The attacker receives the result of the failed log-in and then continuously tries to log in using a random password with the stolen user ID. If the aforementioned sequence is repeated and the log-in fails 2 more times, the user ID is blocked by the security agent. In addition, the security agent requests fingerprint authentication to unblock the user ID from the device enrolled in the HBDP. As a result, platform users will be able to recognize that there has been an illegal log-in attempt. Furthermore, the security agent forwards these attempts to the platform administrator to help them analyze illegal log-in attempts based on the distributed ledger in detail.

#### Prevention of the Misuse (or Abuse) of eHealth Data by an Unauthorized User

**Figure 15:**
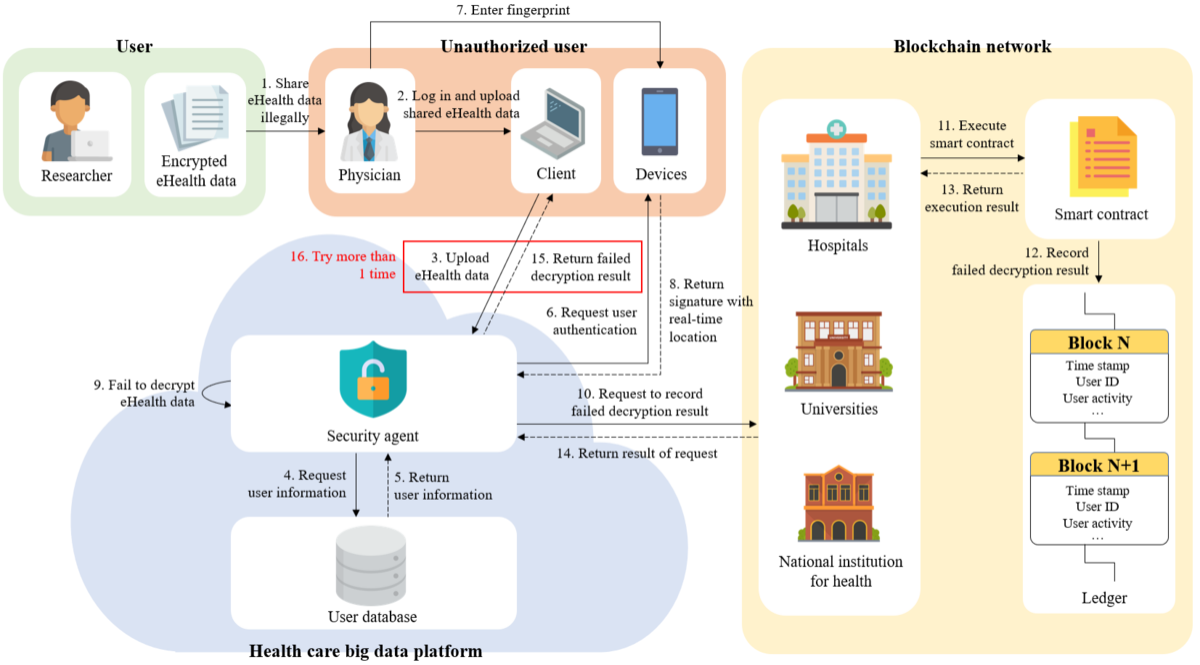
Detection of unauthorized user.

The eHealth data that can be downloaded from the HBDP depend on the user’s department and position. For this reason, there is a possibility that unauthorized users can receive encrypted eHealth data from an authorized user. Therefore, we assume that an unauthorized user of the eHealth data wants to use illegally shared or leaked eHealth data. Figure 15 shows the procedure for detecting the illegal sharing of eHealth data. The details are described in the following paragraphs.

The unauthorized user first obtains encrypted eHealth data in the wrong way from the authorized user. After that, the unauthorized user connects to the HBDP and uploads the illegally shared eHealth data to be used after log-in to the platform. The security agent that receives the eHealth data obtains user information from the user DB and requests fingerprint authentication from the enrolled device. The unauthorized user performs fingerprint authentication. If authentication is successful, the device sends a real-time location along with the signature to the security agent. The security agent then decrypts the eHealth data with the received attributes and real-time location after verification of the signature. However, the decryption of eHealth data fails because the unauthorized user’s attributes do not match the user attributes used for the encryption of the eHealth data. The security agent requests that the decryption result be recorded on the distributed ledger and then informs the unauthorized user that the decryption has failed after the unauthorized user’s activity is recorded on the ledger. If the aforementioned sequence is repeated and the decryption fails one more time, the user ID is blocked by the security agent. The security agent also sends these attempts to the platform administrator to help them analyze illegal use attempts based on the ledger in detail.

In our implementation, various security threats can be detected and blocked, as can unauthenticated and unauthorized users. For example, even if attackers try to decrypt the eHealth data by stealing the user’s ID and password, decryption is impossible as fingerprint authentication fails. In addition, even if fingerprint authentication is successful by manipulating the device, decryption is impossible because of incorrect real-time location information. In other words, the HBDP has ensured the security and privacy of eHealth data. Furthermore, the platform administrator can detect illegal users through periodical monitoring as all user activities on the HBDP are recorded in the distributed ledger. In particular, to use even leaked data, they should be decrypted on the HBDP depending on the use phase. Thus, the administrator can detect this behavior through monitoring. Finally, the HBDP also does not ensure the usefulness of eHealth data via column-level encryption even if leaks by malicious users occur. In conclusion, the HBDP can provide researchers with an open and secure environment in which to efficiently analyze eHealth data.

### Performance Evaluation of the HBDP

#### Overview

Our main concepts are the proposal of a secure research platform for health care and the detection of illegal users using the distributed ledger. For this concept, we presented case studies in the previous section. However, performance is an important factor in proving system efficiency, so we briefly present and describe a performance evaluation of the implemented HBDP in this section.

#### Average Time for Cryptography

To measure the average time, we performed 10 rounds of encryption and decryption with changes in the number of rows and sensitive columns, as shown in Textbox 7.

**Figure 16:**
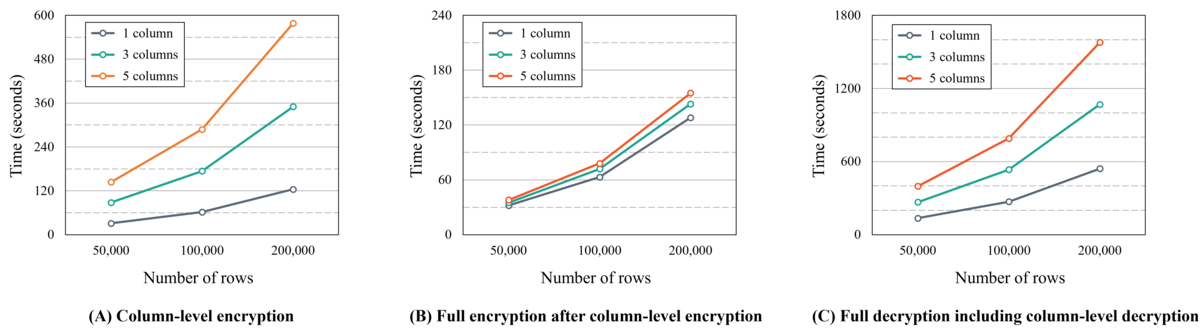
Average time of cryptography.

Figure 16 shows the average encryption and decryption times for the number of rows per number of sensitive columns. Figure 16A shows the average column-level encryption time based on the number of rows. In Figure 16A, if the maximum number of rows is 200,000 and the number of sensitive columns is 5, the maximum average time is approximately 10 minutes. Furthermore, Figure 16B shows the average full encryption time for changes in the number of rows versus each number of encrypted sensitive columns when the user downloads eHealth data from the platform (see the Download Phase section). Significantly, as this work encrypts the rows, the number of encrypted sensitive columns does not greatly affect the full encryption time. In other words, the encryption time is not dramatically increased with an increase in the number of encrypted sensitive columns. Figure 16C shows the average full decryption including column-level decryption time for the number of rows versus each number of encrypted sensitive columns when the user uses eHealth data on the HBDP (see the Use Phase section). As this work performs decryption twice, the decryption time required increases dramatically compared with full encryption. If the maximum number of rows is 200,000 and the number of sensitive columns is 5, the average time is approximately 27 minutes, so the HBDP has limitations in use for actual cases. However, we believe that several methods can solve this problem. A discussion of these methods is detailed in the following subsections.

**Simulation parameters for evaluation of cryptography.**

**Simulation parameters**

- Rounds: 10
- Number of rows: 50,000, 100,000, and 200,000
- Number of sensitive columns within 46 columns: 1, 3, and 5
- Type of cryptography: column-level encryption, full encryption after column-level encryption in the download phase, and full decryption including column-level decryption in the use phase

#### Blockchain Performances

The private blockchain is a distributed logging system that helps detect illegal users on the HBDP. For this reason, we did not evaluate the block and query times and focused only on the accountability and nonrepudiation provided by blockchain features. Therefore, in this section, the write and read times of the designed smart contract are evaluated using Hyperledger Caliper (The Linux Foundation) [44]. We first executed 5 rounds of writing transactions onto the ledger of the blockchain network, with 1000 transactions in each round at rates of 100, 150, 200, 250, and 300 transactions per second (TPS), as shown in Textbox 8. We then executed 5 rounds of reading transactions into the ledger’s blockchain network at rates of 100, 150, 200, 250, and 300 TPS, with 1000 transactions in each round after writing 100 transactions. In particular, at this time, we assume that the platform administrator searches 100 records of previous user activity.

**Figure 17:**
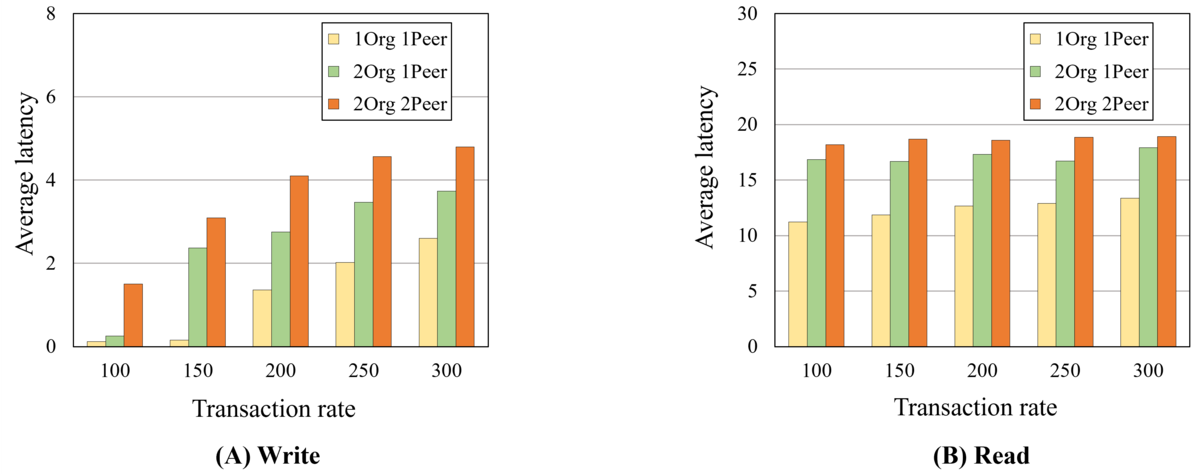
Average transaction latencies.

**Figure 18:**
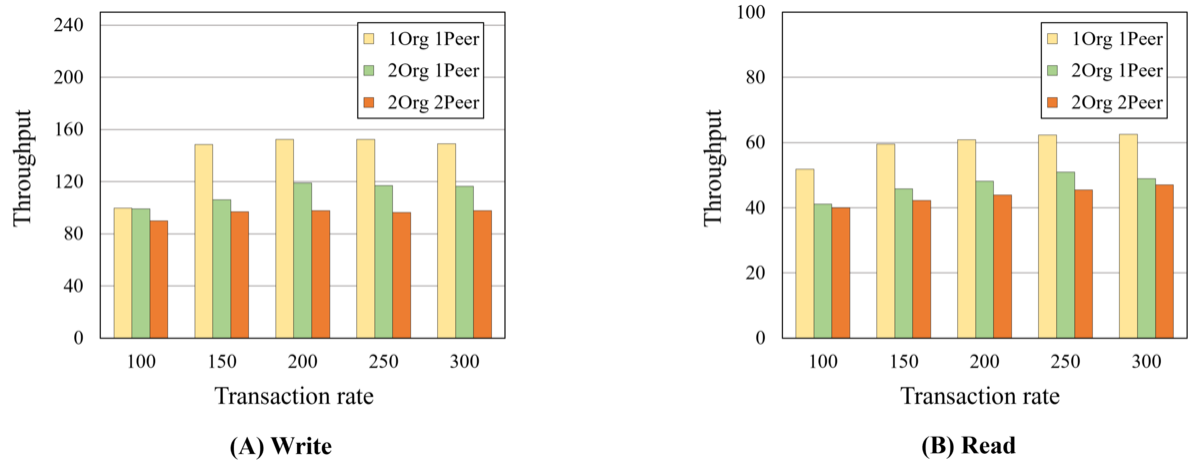
Transaction throughputs.

Figure 17 and Figure 18 show the average latencies and throughputs of our executions. In Figure 17A, the 1Org with 1Peer in write mode takes <3 seconds in 300 TPS, which is a much lower latency than other networks. Conversely, the 1Org with 1Peer in write mode has a higher throughput (approximately 150 TPS) than other networks, as shown in Figure 18A. The 1Org with 1Peer in read mode has an average latency of approximately 14 seconds and a throughput of approximately 63 TPS in 300 TPS, as shown in Figure 17B and Figure 18B. For the 2Orgs with 2Peers in read mode, the average latency and throughput reach approximately 19 seconds and 47 TPS, respectively, in 300 TPS. The results show that many organizations and peers reached high latency and low throughput in both read and write modes, so the latency and throughput are inversely proportional in write mode.

**Simulation parameters for evaluation of the blockchain.**

**Simulation parameters**

- Rounds: 5
- Transactions: 1000 each
- Transaction rates: 100, 150, 200, 250, and 300
- Transaction mode: read and write
- Networks: 2Orgs with 2Peers, 2Orgs with 1Peer, and 1Org with 1Peer
- Orderer: solo

## Discussion

### Principal Findings

In the Results section, we first conducted a security analysis of the HBDP. The results showed that the HBDP provides a secure environment. We then presented the average times for cryptography and blockchain performance of the HBDP to evaluate its efficiency. As a result, some performances (eg, high decryption time depending on the number of encrypted columns) showed to need improvement. Therefore, we first discuss these results in this section. Some limitations of the HBDP are then presented. In addition, we compare the HBDP with prior works. Finally, we describe our further works on blockchain and IoT.

The HBDP provides a secure research environment but has several challenges to solve to be efficient. Thus, this subsection discusses our results and these challenges in detail. First, to prove accountability in the HBDP, we presented case studies on detecting unauthenticated and unauthorized users via the implemented HBDP. In addition, we described some methods to detect other security threats via the process of access control and monitoring. The results showed that the HBDP supports accountability in access control.

Second, as the number of sensitive columns increased, column-level encryption and full decryption including column-level decryption times increased significantly in our results. This issue would cause inconvenience to users in a real environment. To address this issue, we present some solutions. First, when eHealth data have many sensitive columns, efficient cryptography times can be achieved by merging these columns into 1 column to perform encryption and decryption. Second, the security agent can be configured first to perform decryption of some columns to show eHealth data and then decrypt other columns in the background process. This method may make the user feel that the delay is minimal compared with the previous approach. Finally, in the cloud environment, multiple security agents can be configured to perform parallel processing [45-47]. This method is efficient and the most widely used approach. Unfortunately, our work did not use these methods, but they are expected to provide better cryptography times.

Finally, the read mode in blockchain has higher latency and lower throughput than the write mode by approximately ≥60%. In general, blockchain performance has higher latency and lower throughput in write mode than in read mode, but our evaluation showed the opposite result. This result might have been due to the process of looking up and reading all the records of 100 transactions. Nevertheless, the blockchain can be used sufficiently as a distributed logging system for the HBDP as it did not show poor performance. To prove this effectiveness, our discussion can be extended through a performance comparison evaluation with existing studies. However, we did not conduct the performance comparison because of differences in the implementation environment and configuration. The blockchain performance is generally affected by the role configuration of peers and orderers, differences in consensus algorithms, and blockchain type. Furthermore, even if a smart contract performs the same function, the performance can vary depending on how the smart contract is implemented. In conclusion, we consider that a comparison of the performances is useless in perfect nonequivalent environments.

### Limitations

A health care research platform must offer an efficient environment as the primary purpose of the HBDP is to analyze eHealth data and then use the resulting values for research. For this environment, the interoperability of eHealth data, analytical and visualization tools, and data linkage are needed, but the HBDP implemented a few functions for eHealth data. Hence, in our future work, the HBDP will offer an efficient research platform that provides various analytics and visualization tools (eg, Hadoop, Tableau, and Spark) as software as a service.

Furthermore, in this study, the HBDP only focused on three SRs (ie, access control, encryption of stored eHealth data, and accountability), but various additional SRs (eg, deidentification) are needed for a more secure environment. In addition, even after deidentification of eHealth data, the possibility of reidentification remains. Thus, our future work should also provide other SRs and methods to reduce the risk of reidentification through reidentification assessments in advance [48,49].

Finally, the main scope of this study was access control and accountability for a research platform for health care, so the HBDP did not ensure the integrity and availability of eHealth data. In the HBDP, by also writing the hash of eHealth data on the distributed ledger, integrity can be ensured but not completely. For this reason, the HBDP needs solutions to ensure the integrity and availability of eHealth data for a complete research platform for health care. Availability and integrity can be generally ensured by existing cryptography technologies (eg, diverse types of firewalls, message authentication codes, intrusion detection systems, and hash functions). We also consider that some studies [50-54] are helpful for an efficient health care research platform.

### Comparison With Prior Work

For discussions of the HBDP, this section compares the HBDP with the existing health care research platforms based on defined SRs. Table 2 shows that the HBDP and previous research platforms met specific SRs. First, access control (ie, authentication and authorization methods) was partially addressed in all studies [29-34] and was also addressed in the HBDP. In particular, some of the studies [31,32,34] granted access to eHealth data through direct approval from the relevant authorities or contracts. However, this access control is cumbersome and complex, and there is a possibility of overly limiting the use of data. Some studies [29,34] used RBAC for access control, but RBAC cannot easily provide fine-grained access control. By contrast, ABE encryption generally achieves fine-grained access control as administrators can create detailed security policies using various attributes [55-57]. ABE has also been able to achieve flexible access control recently [58,59]. Hence, the HBDP enables fine-grained access control through the various sensing data of IoT devices as we use ABE encryption, unlike existing research platforms for health care. Furthermore, two studies that provided encryption for stored eHealth data were those by Lunn et al [30], Jones et al [34], and the HBDP. This encryption is needed to prevent illegal leaks by insiders and malicious attackers. Even anonymized eHealth data must be encrypted when they are stored on a research platform to make reidentification difficult and useless if the eHealth data are leaked. Finally, accountability is an audit trail that helps the platform administrator take appropriate action when a security incident occurs and mitigate security threats via monitoring. However, the logging system was implemented in a centralized form in the studies by Lunn et al [30] and Jones et al [34]. A centralized system has difficulty operating a logging service when the system is unavailable, and there is a possibility that the integrity of logs can be undermined by attackers. The HBDP uses a logging system in a decentralized form via blockchain and, thus, even if 1 node is unavailable, logging is still possible and ensures the integrity of logs as all peers own the distributed ledger. Moreover, we presented detailed methods of illegal user detection to prove accountability in the HBDP, unlike previous platforms.

**Table 2:** Comparison of the health care big data platform (HBDP) and related studies.

SRs^a^		Studies						
		Ozaydin et al [29]	Lunn et al [30]	Ashfaq et al [31]	Conde et al [32]	De Moor et al [33]	Jones et al [34]	Ours
**SR 1^b^**								
	Authentication		✓		✓	✓	✓	✓
	Authorization	✓		✓	✓	✓	✓	✓
	Fine-grained access control							✓
SR 2^c^—encryption (encryption level and content of eHealth data)			✓ (Full data and medical report)				✓ (Sensitive data and identifiable data)	✓ (Sensitive data and medical condition)
**SR 3^d^**								
	Decentralization							✓
	Centralization		✓				✓	
	Illegal user detection methods							✓

^a^SR: security requirement.

^b^Access control.

^c^Encryption of stored eHealth data.

^d^Accountability.

### Private Blockchain

eHealth data subjects hope to strengthen their rights by participating directly in eHealth data access decisions. They are also concerned with the privacy and security of eHealth data. However, when they directly participate in access decisions, it has the potential to stifle or unduly limit the usability of eHealth data in research (eg, the approval of researchers’ requests to use eHealth data is delayed for a long time or they are unconditionally refused). Therefore, the subjects’ rights must be ensured in other ways. With the distributed ledger of the blockchain, we expect that providers can supervise although not directly participate in access decisions. For example, eHealth data subjects can easily search the use history and users of their eHealth data at any time via the distributed ledger on the platform and object to the use if there are any issues. In addition, as the recorded history of the blockchain is difficult to alter, it is expected to elicit greater trust from eHealth subjects. Although we did not implement this supervisory function, we expect that further research will help address concerns about the use of eHealth data as well as advance the rights of eHealth data subjects.

### Interoperability on IoT Devices

The eHealth data from various sensors and IoT devices are rapidly increasing and being collected in many health facilities. These eHealth data can improve public health and provide high-quality customized health care services when they are used in research, so they must be offered on a health care research platform. In general, IoT devices are connected to and managed by IoT platforms. However, it is currently difficult to share or use collected eHealth data because of the various interoperability issues on IoT platforms. In particular, secure interoperability cannot be guaranteed as each IoT platform has different access control methods and security policies for IoT devices. Therefore, our future research will provide various and detailed eHealth data to researchers by ensuring the secure interoperability of IoT platforms on the HBDP.

### Conclusions

The use of eHealth data in health care research offers promising potential and advantages. However, eHealth data are more sensitive than other big data as they contain more personal information, so the privacy and security of eHealth data must be ensured for them to be used in studies. In addition, eHealth data subjects are still concerned about unauthorized data reuse and sharing within existing health care research platforms. Thus, we designed a more secure collaborative platform for health care research called the HBDP. This platform ensures the privacy and security of eHealth data using a private blockchain and ABE cryptography. The private blockchain operates as a decentralized persistent log DB in which all activities occurring on the HBDP are recorded with time stamps. As a result, the records in the blockchain (ie, distributed ledger) help platform administrators and users detect and respond to unauthenticated and unauthorized users on the HBDP. ABE cryptography ensures privacy even if eHealth data are leaked from the platform and enables detailed and fine-grained access control using situational information. Furthermore, we developed and tested the HBDP, blockchain network, and an Android app using Hyperledger Fabric, OpenStack, and OpenABE library to show the feasibility of the platform. We also described the detection of illegal users (ie, unauthenticated and unauthorized users) via case studies. As this study focused only on a secure environment for health care research, some future work is needed to provide an efficient and complete research platform. Nevertheless, we believe that the HBDP will provide a sufficiently secure environment for the use of eHealth data in health care research.

## Abbreviations

ABE: attribute-based encryption
CDA: Clinical Document Architecture
CDM: Common Data Model
DARS: Data Access Request Service
DB: database
DICOM: Digital Imaging and Communications in Medicine
EMR: electronic medical record
FHIR: Fast Healthcare Interoperability Resources
GDPR: General Data Protection Regulation
HBDP: health care big data platform
HL7: Health Level 7
HRADIS: Healthcare Research and Analytics Data Infrastructure Solution
IoT: Internet of Things
NHS: National Health Service
OMOP: Observational Medical Outcomes Partnership
PHR: personal health record
RBAC: role-based access control
SR: security requirement
TPS: transactions per second
XDS: Cross-Enterprise Document Sharing
